# Performance of a novel *KRAS* mutation assay for formalin-fixed paraffin embedded tissues of colorectal cancer

**DOI:** 10.1186/2193-1801-4-7

**Published:** 2015-01-05

**Authors:** Kazuko Sakai, Azusa Yoneshige, Akihiko Ito, Yoji Ueda, Satoshi Kondo, Hitoshi Nobumasa, Yoshihiko Fujita, Yosuke Togashi, Masato Terashima, Marco A De Velasco, Shuta Tomida, Kazuto Nishio

**Affiliations:** 1 Department of Genome Biology, Kinki University Faculty of Medicine, Osaka-Sayama, Osaka, Japan; 2 Department of Pathology, Kinki University Faculty of Medicine, Osaka-Sayama, Osaka, Japan; 3 New Projects Development Division, Toray Industries, Inc., Kamakura, Kanagawa, Japan

**Keywords:** Colorectal cancer, *KRAS* mutation, Anti-EGFR antibody, 3D-Gene® *KRAS* mutation assay, Companion diagnosis

## Abstract

We compared the performance of the 3D-Gene® mutation assay (3D-Gene® *KRAS* mutation assay kit) with the Scorpion-ARMS (therascreen® KRAS RGQ PCR Kit) and Luminex (MEBGEN™ KRAS kit) assays for the detection of *KRAS* mutations in formalin-fixed, paraffin-embedded tissue samples from 150 patients diagnosed with colorectal cancer. DNA was extracted from the paraffin-embedded tissue samples with or without macrodissection under hematoxylin and eosin staining and the *KRAS* mutation status was independently determined using these assays. Discordant results were re-analyzed by Sanger sequencing. Mutation detection analysis was successfully performed in all 150 specimens using the 3D-Gene® mutation assay without an invalid case. The concordance rate between the 3D-Gene® mutation assay and Scorpion-ARMS or Luminex was 98.7% (148/150). *KRAS* mutations were detected at a frequency of 35.3% (53/150) in colorectal cancer specimens. Three discrepant cases were found between the three assays. Overall, our results demonstrate a high concordance rate of between the 3D-Gene® mutation assay and the two existing *in-vitro* diagnostics kits. All three assays proved to be validated methods for detecting clinically significant *KRAS* mutations in paraffin-embedded tissue samples.

**Electronic supplementary material:**

The online version of this article (doi:10.1186/2193-1801-4-7) contains supplementary material, which is available to authorized users.

## Background

Several studies have shown that cetuximab and panitumumab do not improve progression-free or overall survival for advanced colorectal cancer patients with mutated *KRAS* (Karapetis et al. 2008; Amado et al. 2008; De Roock et al. 2008; Lièvre et al. 2006, 2008). KRAS is a GTP binding protein with a molecular weight of 21 kDa. KRAS is activated by GTP binding triggered by upstream signals such as EGFR. The *KRAS* gene is located on chromosome 12 and consists of 4 exons and 3 introns. Single base mutations of *KRAS* genes decrease GTPase activity and leads to constitutive activation of KRAS. The frequency of *KRAS* mutations is 34.6% in colorectal cancer, according to the COSMIC (Catalogue of Somatic Mutation in Cancer) database. Comparable frequencies have also been reported in population–based studies. (Andreyev et al. 1998; Karapetis et al. 2008). Major mutations of *KRAS* occur in exon 2 (codons 12 and 13). Two phase III clinical trials, OPUS and CRYSTAL, have demonstrated that codon 12 and 13 mutations are predictive for cetuximab activity in combination with FOLFOX or FOLFIRI, respectively (Bokemeyer et al. 2011; Van Cutsem et al. 2011). An additional study also demonstrated that cetuximab combined with first line chemotherapy is effective for patients with *KRAS* G13D tumor mutations (Bokemeyer et al. 2012). A variety of studies have shown that *KRAS* mutations influence the response to the anti-EGFR antibodies cetuximab and panitumumab (Jonker et al. 2007; Karapetis et al. 2008). Several methods have been reported for the detection of *KRAS* mutations in formalin-fixed paraffin embedded (FFPE) tissues (Gonzalez de Castro et al. 2012; Chang et al. 2013; Altimari et al. 2013). The Sanger sequencing method is currently the gold standard (Allegra et al. 2009), however, several *KRAS* mutation kits have become available for colorectal cancer patients.

3D-Gene® is a microarray technology developed by Toray. This technology has been applied for gene expression profiling and miRNA analysis (Sato et al. 2009; Sudo et al. 2012). 3D-Gene® microarray has a micro-columnar structure and uses a bead-agitation technique to achieve high sensitivity and reproducibility. Toray has established a new 3D-Gene® assay for the detection of *KRAS* mutations in tumor tissues using the PCR-rSSO (PCR-reverse sequence-specific oligonucleotide) method. Both Luminex and 3D-Gene® assays are based on the PCR-rSSO method. However, fluorescent beads are detected by flow cytometry in the Luminex assay. On the other hand, the 3D-Gene® assay utilizes array technology for the detection of the fluorescent PCR product. In this study, we compared the clinical performance of the 3D-Gene® mutation assay with two other assays (Scorpion-ARMS and Luminex) using FFPE tissue specimens from colorectal cancer patients (Harlé et al. 2013; Fukushima et al. 2011).

## Results

### Sensitivity of 3D-Gene® *KRAS*mutation assay

The minimum number of mutant DNA copies that were reliably detected by the 3D-Gene® assay was 350–700 copies (2.5-5%) as determined by using serially diluted mutant DNA (G12S) against wild-type DNA of HeLa cells (15000 copies; 50 ng). The minimum threshold for the number of mutant DNA copies was 700–1400 (5-10%) using serially diluted mutant DNA (G13D) against wild-type DNA of HeLa cells.

### Study population

One hundred fifty FFPE tissue samples from patients with colorectal cancer in Kinki University Hospital, Faculty of Medicine were examined. All of the samples were available for analysis. The clinical and pathological characteristics are summarized in Additional file 1: Table S1.

### Mutation types of *KRAS*

*KRAS* mutations were detected in 53/150 (36.0%), 53/150 (35.3%) and 51/150 (34.0%) cases by 3D-Gene®, Scorpion-ARMS and Luminex, respectively. (Additional file 1: Table S2). Mutations were mainly located in codon 12 of *KRAS* (40/53, 75.5%). G12V mutations were most frequently observed by all assays (14/53, 26.4%). G13D was detected in 13 cases, but no other mutation in codon 13 was detected.

### Invalid test rate

Mutation analysis of exons 12–13 of the *KRAS* gene was successfully performed in all 150 specimens (100%) using the 3D-Gene® mutation assay. No invalid test results were detected in the two other assays. The invalid test rate for this study was 0%.

### Method correlation agreement analysis

We compared the *KRAS* mutation status across the three assays: 3D-Gene®, Scorpion-ARMS, and Luminex. The correlation rates between 3D-Gene® and Scorpion-ARMS or 3D-Gene® and Luminex were 98.7% (148/150). The 3D-Gene® assay detected mutant *KRAS* in two specimens, but were wild-type by the Scorpion-ARMS method. Two specimens had a discrepant status between 3D-Gene® and Luminex. The first specimen had a wild-type *KRAS* by 3D-Gene® but was mutant by Luminex. Second specimen had a mutant *KRAS* by 3D-Gene® but was wild-type by Luminex. The concordance of the data from the three *KRAS* mutation assays is shown in Additional file 1: Tables S3 and S4. Only three discordant results between the three *KRAS* mutation assays were observed. The site specific concordant rates are summarized in Tables 1 and 2. Inconsistencies were found in two mutations (G12C and G13D), while others remained completely matched. No statistically significant differences were observed between the correlations of 3D-Gene® and Scorpion-ARMS (κ = 0.97, 95% CI 0.86 – 1.000 and 3D-Gene® and Luminex (κ = 0.97, 95% CI 0.86 – 1.00).

**Table 1:** **Detailed concordance rate between 3D-Gene® and Scorpion-ARMS**

Mutation	MD concordance	MND concordance	Total concordance
G12A	100% (2/2)	100% (148/148)	100% (150/150)
G12C	88.3% (5/6)	99.3% (143/144)	98.6% (148/150)
G12D	100% (13/13)	100% (137/137)	100% (150/150)
G12R	100% (3/3)	100% (147/147)	100% (150/150)
G12S	100% (3/3)	100% (147/147)	100% (150/150)
G12V	100% (14/14)	100% (136/136)	100% (150/150)
G13D	92.3% (12/13)	99.3% (137/138)	99.3% (149/150)

MD: mutation detected, MND: mutation not detected.

**Table 2:** **Detailed concordance rate between 3D-Gene® and Luminex**

Mutation	MD concordance	MND concordance	Total concordance
G12A	100% (2/2)	100% (148/148)	100% (150/150)
G12C	100% (5/5)	99.3% (144/145)	99.3% (149/150)
G12D	100% (13/13)	100% (137/137)	100% (150/150)
G12R	100% (3/3)	100% (147/147)	100% (150/150)
G12S	100% (3/3)	100% (147/147)	100% (150/150)
G12V	100% (14/14)	100% (136/136)	100% (150/150)
G13D	100% (13/13)	100% (137/137)	100% (150/150)

MD: mutation detected, MND: mutation not detected.

### Re-analysis of discordance by Sanger sequencing

Discordant results between the three assays were identified in three cases. Discordances were observed between G12C or wild-type *KRAS* in two cases, and between G13D and wild-type *KRAS* in one case. The three discordant cases were retested using Sanger sequencing on DNA samples extracted for the 3D-Gene® mutation assay. A mutant allele was not detected by Sanger sequencing.

## Discussion

*KRAS* mutation testing has become mandatory prior to the administration of therapy with the anti-EGFR antibodies, cetuximab or panitumumab, for patients with advanced colorectal cancer. *KRAS* mutation screening based on the Scorpion-ARMS *KRAS* mutation assay (therascreen® KRAS RGQ PCR Kit, Qiagen) has been approved for *in vitro* diagnostic use in Japan. A kit using Luminex (MEBGEN^™^ *KRAS* Mutation Detection Kit, MBL) was also approved (Fukushima et al. 2011). In this study, we examined the feasibility and robustness of the 3D-Gene® *KRAS* mutation assay kit.

The 3D-Gene® KRAS mutation assay kit is designed to detect 12 *KRAS* mutations on codons 12 and 13, and requires 50 ng of DNA. This assay has been validated with samples comprised of a minimum of 2.5% of tumor DNA. Thus, experimental assays demonstrate a sensitivity of 2.5%. This sensitivity is comparable to that of other methods such as Scorpion-ARMS and Luminex. We have demonstrated that the RFU values for mutant probes were remarkably lower than the respective cut-off values using HeLa DNA (wild-type *KRAS*). Based on this study, we believe that this assay provides sufficient specificity for the reliable detection of *KRAS* mutations. Estimated hands on time for Scorpion-ARMS, 3D-Gene®, and Luminex are 2.5, 3, and 3.5 hours, respectively. Assay cost for one sample of 3D-Gene® and Luminex are expected to be lower than that of Scorpion-ARMS when used under the designation for “research use only”.

The correlation between the 3D-Gene® *KRAS* mutation assay and existing *KRAS* mutation assays is high. This result indicates that the 3D-Gene® mutation assay is a robust assay capable of detecting the most common clinically significant *KRAS* mutations and is comparable to existing *KRAS* mutation assays.

We detected a total of 3 discordant results among 150 (2.0%) cases in this study. The concordance rate was 98.6% between both 3D-Gene® and Scorpion-ARMS (148/150) and 3D-Gene® and Luminex (148/150). Re-analysis by Sanger sequencing could not detect any mutations. However, this might be due to tumor heterogeneity. The whole process (from DNA extraction to detection) was performed independently in each of the three assays. Differences between the methods of DNA extraction may have contributed to the discordance among the three assays.

*KRAS* mutations in codons 61 and 146, and *BRAF*, *NRAS*, and *PI3KCA* are also related to resistance to anti-EGFR antibodies (Douillard et al. 2013). Therefore, further studies should be carried out to expand the use of 3D-Gene® technology for the detection of “all RAS” and *PI3KCA* mutations.

## Conclusion

In conclusion, our results demonstrate a high concordance rate of between the 3D-Gene® mutation assay and the two existing *in-vitro* diagnostics kits. All three assays proved to be validated methods for detecting clinically significant *KRAS* mutations in paraffin-embedded tissue samples.

## Methods

### Samples

A series of 150 FFPE archived tissues was obtained from 150 Japanese patients with colorectal cancer at Kinki University Faculty of Medicine (2012–2013). All patients enrolled in the study provided written informed consent for the use of resected tissue. This study was approved by the ethics committee of Kinki University Faculty of Medicine (Authorization Number: 25–167). All samples were processed with the 3D-Gene® *KRAS* mutation, Scorpion-ARMS and Luminex assays. Three samples were processed with Sanger sequencing. All 150 samples were from primary colorectal carcinoma. The patient characteristics are listed in Additional file 1: Table S1.

Genomic DNA from A549, HCT-116, and HeLa cells were obtained from ATCC, Takara Bio Inc. (Shiga, Japan) and New England Biolabs Japan Inc. (Tokyo, Japan), respectively.

### Study design

**Figure 1:**
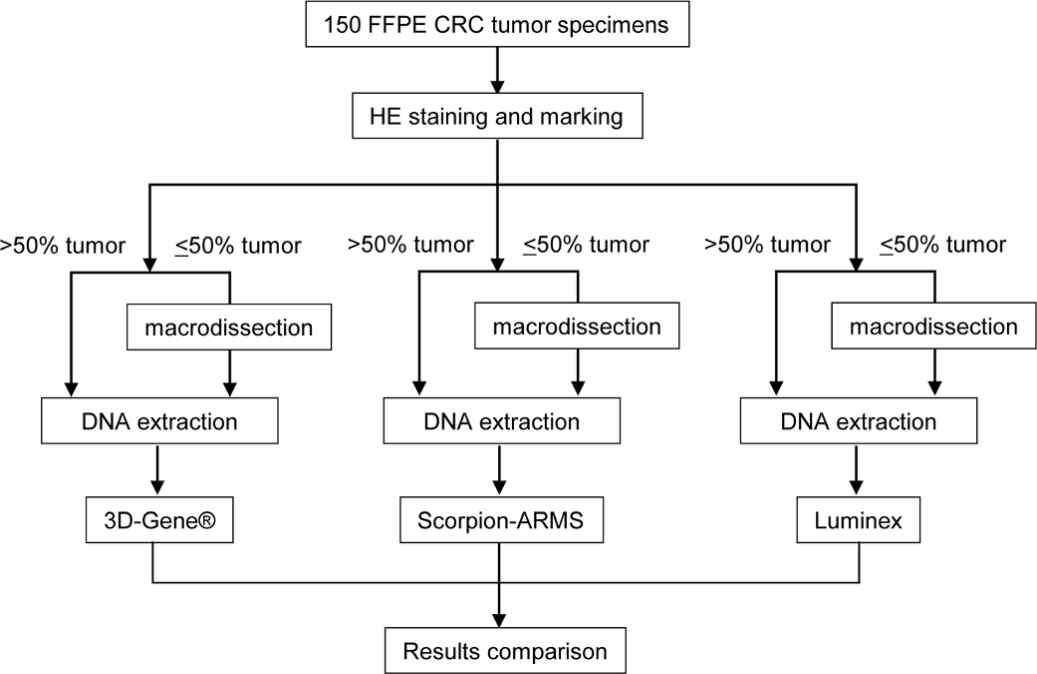
**Study design.** FFPE colorectal cancer tumor specimens (150 cases) were selected and processed using 3D-Gene®, Scorpion-ARMS, and Luminex. The *KRAS* mutation status obtained from the three assays was compared.

The study design is summarized in Figure 1. Colorectal cancer samples (150 cases) were selected by a pathologist. All of the hematoxylin and eosin-stained FFPE slides were examined and the tumor region was marked by a pathologist at Kinki University Faculty of Medicine. If the tumor region represented less than 50% on the tissue slice, the tumor regions were macrodissected. DNA extraction was performed independently for each tissue slice according to the specific standard operating procedure for each of the three assays. The 3D-Gene® *KRAS* mutation assay (Toray, Tokyo, Japan) was performed at the Department of Genome Biology, Kinki University (Osaka, Japan). The Scorpion-ARMS (therascreen® KRAS RGQ PCR Kit, Qiagen, Tokyo) and Luminex (MEBGEN™ *KRAS* gene mutation detection kit, MBL, Nagoya, Japan) assays were performed at the laboratories of LSI-Medience Co. (Tokyo, Japan) and SRL Inc. (Hino, Japan), respectively. All data was compiled and analyzed at Department of Genome Biology, Kinki University.

### DNA extraction

Paraffin blocks were serially cut at 5 μm thickness. DNA was purified from the tissue sections using a QIAamp DNA FFPE Tissue kit (Qiagen, Valencia, CA) according to the manufacturer’s instructions. DNA quantity was determined by NanoDrop spectrophotometry (NanoDrop Technologies, Wilmington, DE).

### 3D-Gene® mutation assay

**Figure 2:**
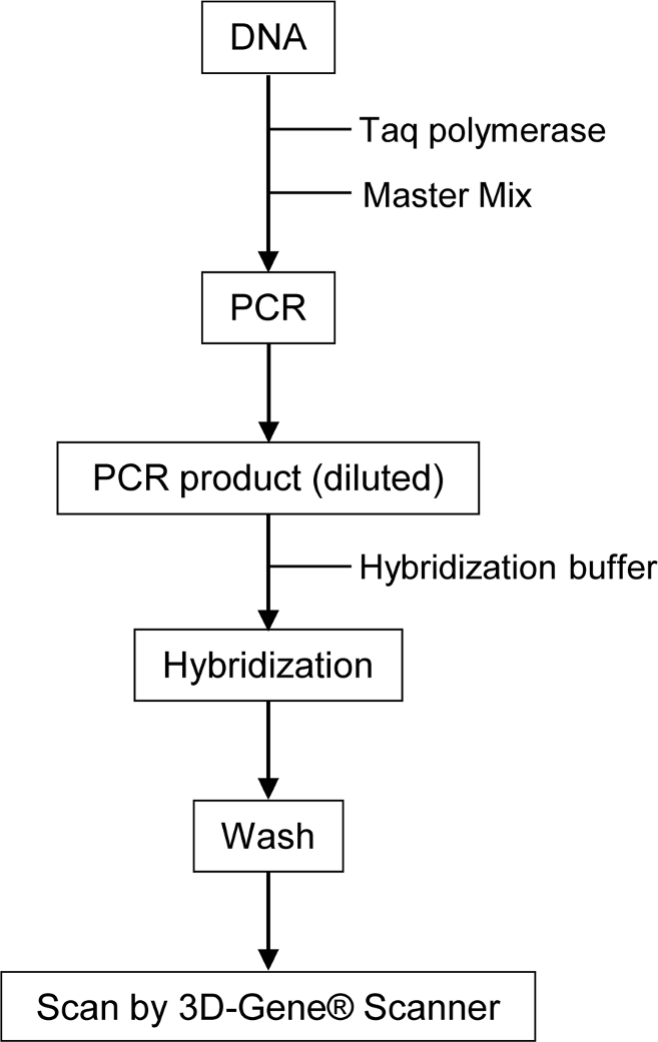
**Assay workflow.** Workflow of multiple *KRAS* mutations by the 3D-Gene® *KRAS* mutation assay.

The 3D-Gene® *KRAS* mutation assay kit (Toray, Tokyo, Japan) was used for *KRAS* mutation profiling. This kit consists of a DNA chip and PCR reagents. The DNA chip has probes to detect 12 mutations of KRAS codon 12 and 13 (G12S, G12C, G12R, G12D, G12V, G12A, G13S, G13C, G13R, G13D, G13V and G13A). The cutoff value for determining positive mutations was set to 300, but G12C and G13C were set 385 and 537, respectively. Fifty nanograms of DNA derived from FFPE tissue was amplified with the supplied reagent. The PCR product labeled with fluorescent dye was hybridized onto the DNA chip surface after denaturation at 95°C for 2 min. Chips were incubated at 59°C for 30 min under agitation at 1,400 rpm. After hybridization, the DNA chips were washed and dried in an ozone-free environment. The fluorescent image was scanned with the 3D-Gene® Scanner 3000 (Toray Industries, Inc.). The images obtained from the DNA chip were quantified by measuring the relative fluorescence unit (RFU) using 3D-Gene® ExTraction software (Toray Industries, Inc.). This assay determined the mutation to be positive if signal detected was greater than the cutoff value. The assay workflow is illustrated in Figure 2. Probe arrangements in a cell and representative scanned image of a microarray are shown in Additional file 2: Figures S1 and S2, respectively. Cutoff values were established using *KRAS* wild-type and mutant plasmid DNA. We set the average + 5SD (RFU) values obtained from the *KRAS* wild-type plasmid DNA as tentative cutoff value. The values for the mutation sites were 385, 537, and 300 RFU for G12C, G13C, and others, respectively. When the 3D-Gene® assay was performed using codon 12 mutant plasmid DNA, the minimum measured value among codon 12 mutation was 1009 RFU (G12R), whereas all codon 13 mutation values were below 300 RFU. When the 3D-Gene® assay was performed using codon 13 mutant plasmid DNA, the minimum measured value among codon 13 mutation was 332 RFU (G13S), whereas all codon 12 mutation values were below 300 RFU. All assays were performed eight times independently. Based on these data, we set the cutoff values for G12C, G13C, and others at 385, 537, and 300 RFU, respectively. Similarly, the cutoff value for wild-type was set to 300 RFU.

### Sensitivity assay

The sensitivity of the 3D-Gene® mutation assay was evaluated by mixing G12S (codon 12) or G13D (codon 13) mutated and wild-type DNA from cell lines (A549 as codon 12 mutated, HCT-116 codon 13 and HeLa cells as wild-type, respectively) at 100%, 50%, 25%, 5%, 2.5% and 1% ratios.

### Scorpion-ARMS

The Scorpion-ARMS assay was analyzed using the therascreen® KRAS RGQ PCR Kit (Qiagen). The Scorpion-ARMS assay is a real time-PCR assay that combines the Amplification Refractory Mutation System (ARMS) and Scorpions fluorescent primer/probe system. This assay detects G12S, G12C, G12R, G12D, G12V, G12A, and G13D mutations of *KRAS*. The sensitivity is 1%. This assay was performed according to the manufacturer’s guidelines (Qiagen). Briefly, DNA was isolated from FFPE tissue samples and the total sample DNA assessed by amplifying a region of exon 2 from *KRAS* by PCR. Next, the DNA samples were tested for the presence or absence of *KRAS* mutations by real-time PCR using a Scorpion probe and primers specific for wild-type and mutant *KRAS* DNA. The differences between the mutation assay cycle threshold (Cγ) values were determined. Samples were designated mutation positive if the δCγ was less than the cutoff δCγ value.

### Luminex

The Luminex assay was analyzed using the MEBGEN^™^ *KRAS* kit (MBL, Nagoya, Japan), which is currently approved for clinical use by the Ministry of Health, Labour and Welfare of Japan (Fukushima et al. 2011). The Luminex assay is based on PCR-rSSO method (Itoh et al. 2005); First, 50 ng of template DNA collected from FFPE tissue samples was amplified by PCR using a biotin-labeled primer. Thereafter, the PCR products and fluorescent Luminex beads (oligonucleotide probes complementary to wild and mutant genes bound to the beads) were hybridized and labeled with streptavidin–phycoerythrin. Subsequently, the products were processed by the Luminex assay and the data collected was then analyzed using UniMAG software (MBL, Japan). This assay detects G12S, G12C, G12R, G12D, G12V, G12A, G13S, G13C, G13R, G13D, G13V and G13A mutations of *KRAS*.

### Sequencing analysis

DNA samples obtained from specimens that were discordant between three assays were amplified using the following site-specific primers: forward 5′ -AAGGCCTGCTGAAAATGACTG- 3′, reverse 5′ -GTCCTGCACCAGTAATATGC- 3′. The purified products were sequenced using BigDye terminator v3.1 (Applied Biosystems, Foster City, CA) with ABI 3100 Genetic Analyzer (Applied Biosystems).

### Statistics

Kappa statistics were used to compare the 3D-Gene® *KRAS* mutation assay with the Scorpion-ARMS and Luminex assays.

## Electronic supplementary material

Additional file 1: Table S1: Clinicopathological characteristics primary colorectal cancer patients providing FFPE samples. **Table S2.** Results of three different assay. **Table S3.** Methods correlation between 3D-Gene® and Scorpion-ARMS. **Table S4.** Methods correlation between 3D-Gene® and Luminex. (XLSX 17 KB)

Additional file 2: Figure S1: Probe arrangements in a microarray cell. The microarray slide is divided into 8 compartments. Each cell has 13 probes of the *KRAS* gene, and each probe is represented six times on the slide. WT: wild-type *KRAS*, NC: negative control. **Figure S2.** Representative scanned image of a microarray. Images shown are representative of images from three mutant (G12C, G12S and G13D), three wild-type (WT), one WT control and one negative control (distilled water) samples. (PDF 70 KB)

## References

[CR1] Allegra CJ, Jessup JM, Somerfield MR, Hamilton SR, Hammond EH, Hayes DF, McAllister PK, Morton RF, Schilsky RL (2009). American Society of Clinical Oncology provisional clinical opinion: testing for KRAS gene mutations in patients with metastatic colorectal carcinoma to predict response to anti-epidermal growth factor receptor monoclonal antibody therapy. J Clin Oncol.

[CR2] Altimari A, de Biase D, De Maglio G, Gruppioni E, Capizzi E, Degiovanni A, D'Errico A, Pession A, Pizzolitto S, Fiorentino M, Tallini G (2013). 454 next generation-sequencing outperforms allele-specific PCR, Sanger sequencing, and pyrosequencing for routine KRAS mutation analysis of formalin-fixed, paraffin-embedded samples. Onco Targets Ther.

[CR3] Amado RG, Wolf M, Peeters M, Van Cutsem E, Siena S, Freeman DJ, Juan T, Sikorski R, Suggs S, Radinsky R, Patterson SD, Chang DD (2008). Wild-type KRAS is required for panitumumab efficacy in patients with metastatic colorectal cancer. J Clin Oncol.

[CR4] Andreyev HJ, Norman AR, Cunningham D, Oates JR, Clarke PA (1998). Kirsten ras mutations in patients with colorectal cancer: the multicenter “RASCAL” study. J Natl Cancer Inst.

[CR5] Bokemeyer C, Bondarenko I, Hartmann JT, de Braud F, Schuch G, Zubel A, Celik I, Schlichting M, Koralewski P (2011). Efficacy according to biomarker status of cetuximab plus FOLFOX-4 as first-line treatment for metastatic colorectal cancer: the OPUS study. Ann Oncol.

[CR6] Bokemeyer C, Van Cutsem E, Rougier P, Ciardiello F, Heeger S, Schlichting M, Celik I, Köhne CH (2012). Addition of cetuximab to chemotherapy as first-line treatment for KRAS wild-type metastatic colorectal cancer: pooled analysis of the CRYSTAL and OPUS randomised clinical trials. Eur J Cancer.

[CR7] Chang SC, Denne J, Zhao L, Horak C, Green G, Khambata-Ford S, Bray C, Celik I, Van Cutsem E, Harbison C (2013). Comparison of KRAS genotype: therascreen assay vs. LNA-mediated qPCR clamping assay. Clin Colorectal Cancer.

[CR8] De Roock W, Piessevaux H, De Schutter J, Janssens M, De Hertogh G, Personeni N, Biesmans B, Van Laethem JL, Peeters M, Humblet Y, Van Cutsem E, Tejpar S (2008). KRAS wild-type state predicts survival and is associated to early radiological response in metastatic colorectal cancer treated with cetuximab. Ann Oncol.

[CR9] Douillard JY, Oliner KS, Siena S, Tabernero J, Burkes R, Barugel M, Humblet Y, Bodoky G, Cunningham D, Jassem J, Rivera F, Kocákova I, Ruff P, Błasińska-Morawiec M, Šmakal M, Canon JL, Rother M, Williams R, Rong A, Wiezorek J, Sidhu R, Patterson SD (2013). Panitumumab-FOLFOX4 treatment and RAS mutations in colorectal cancer. N Engl J Med.

[CR10] Fukushima Y, Yanaka S, Murakami K, Abe Y, Koshizaka T, Hara H, Samejima C, Kishi Y, Kaneda M, Yoshino T (2011). High-throughput screening method of KRAS mutations at codons 12 and 13 in formalin-fixed paraffin-embedded tissue specimens of metastatic colorectal cancer. Gan To Kagaku Ryoho.

[CR11] Gonzalez de Castro D, Angulo B, Gomez B, Mair D, Martinez R, Suarez-Gauthier A, Shieh F, Velez M, Brophy VH, Lawrence HJ, Lopez-Rios F (2012). A comparison of three methods for detecting KRAS mutations in formalin-fixed colorectal cancer specimens. Br J Cancer.

[CR12] Harlé A, Busser B, Rouyer M, Harter V, Genin P, Leroux A, Merlin JL (2013). Comparison of COBAS 4800 KRAS, TaqMan PCR and high resolution melting PCR assays for the detection of KRAS somatic mutations in formalin-fixed paraffin embedded colorectal carcinomas. Virchows Arch.

[CR13] Itoh Y, Mizuki N, Shimada T, Azuma F, Itakura M, Kashiwase K, Kikkawa E, Kulski JK, Satake M, Inoko H (2005). High-throughput DNA typing of HLA-A, -B, -C, and -DRB1 loci by a PCR-SSOP-Luminex method in the Japanese population. Immunogenetics.

[CR14] Jonker DJ, O’Callaghan CJ, Karapetis CS, Zalcberg JR, Tu D, Au HJ, Berry SR, Krahn M, Price T, Simes RJ, Tebbutt NC, van Hazel G, Wierzbicki R, Langer C, Moore MJ (2007). Cetuximab for the treatment of colorectal cancer. N Engl J Med.

[CR15] Karapetis CS, Khambata-Ford S, Jonker DJ, O’Callaghan CJ, Tu D, Tebbutt NC, Simes RJ, Chalchal H, Shapiro JD, Robitaille S, Price TJ, Shepherd L, Au HJ, Langer C, Moore MJ, Zalcberg JR (2008). K-ras mutations and benefit from cetuximab in advanced colorectal cancer. N Engl J Med.

[CR16] Lièvre A, Bachet JB, Le Corre D, Boige V, Landi B, Emile JF, Côté JF, Tomasic G, Penna C, Ducreux M, Rougier P, Penault-Llorca F, Laurent-Puig P (2006). KRAS mutation status is predictive of response to cetuximab therapy in colorectal cancer. Cancer Res.

[CR17] Lièvre A, Bachet JB, Boige V, Cayre A, Le Corre D, Buc E, Ychou M, Bouché O, Landi B, Louvet C, André T, Bibeau F, Diebold MD, Rougier P, Ducreux M, Tomasic G, Emile JF, Penault-Llorca F, Laurent-Puig P (2008). KRAS mutations as an independent prognostic factor in patients with advanced colorectal cancer treated with cetuximab. J Clin Oncol.

[CR18] Sato F, Tsuchiya S, Terasawa K, Tsujimoto G (2009). Intra-platform repeatability and inter-platform comparability of microRNA microarray technology. PLoS One.

[CR19] Sudo H, Mizoguchi A, Kawauchi J, Akiyama H, Takizawa S (2012). Use of non-amplified RNA samples for microarray analysis of gene expression. PLoS One.

[CR20] Van Cutsem E, Köhne CH, Láng I, Folprecht G, Nowacki MP, Cascinu S, Shchepotin I, Maurel J, Cunningham D, Tejpar S, Schlichting M, Zubel A, Celik I, Rougier P, Ciardiello F (2011). Cetuximab plus irinotecan, fluorouracil, and leucovorin as first-line treatment for metastatic colorectal cancer: updated analysis of overall survival according to tumor KRAS and BRAF mutation status. J Clin Oncol.

