# Supplementary material for: Performance of a novel KRAS mutation assay for formalin-fixed paraffin embedded tissues of colorectal cancer
Source: Springerplus. 2015 Jan 5;4(1):7. doi: 10.1186/2193-1801-4-7 (PMC4320212; doi:10.1186/2193-1801-4-7)
Supplement: Supplementary file 2 — Additional file 2: Figure S1: Probe arrangements in a microarray cell. The microarray slide is divided into 8 compartments. Each cell has 13 probes of the KRAS gene, and each probe is represented six times on the slide. WT: wild-type KRAS, NC: negative control. Figure S2. Representative scanned image of a microarray. Images shown are representative of images from three mutant (G12C, G12S and G13D), three wild-type (WT), one WT control and one negative control (distilled water) samples. (PDF 70 KB) [file 40064_2014_1507_MOESM2_ESM.pdf]

Figure S1

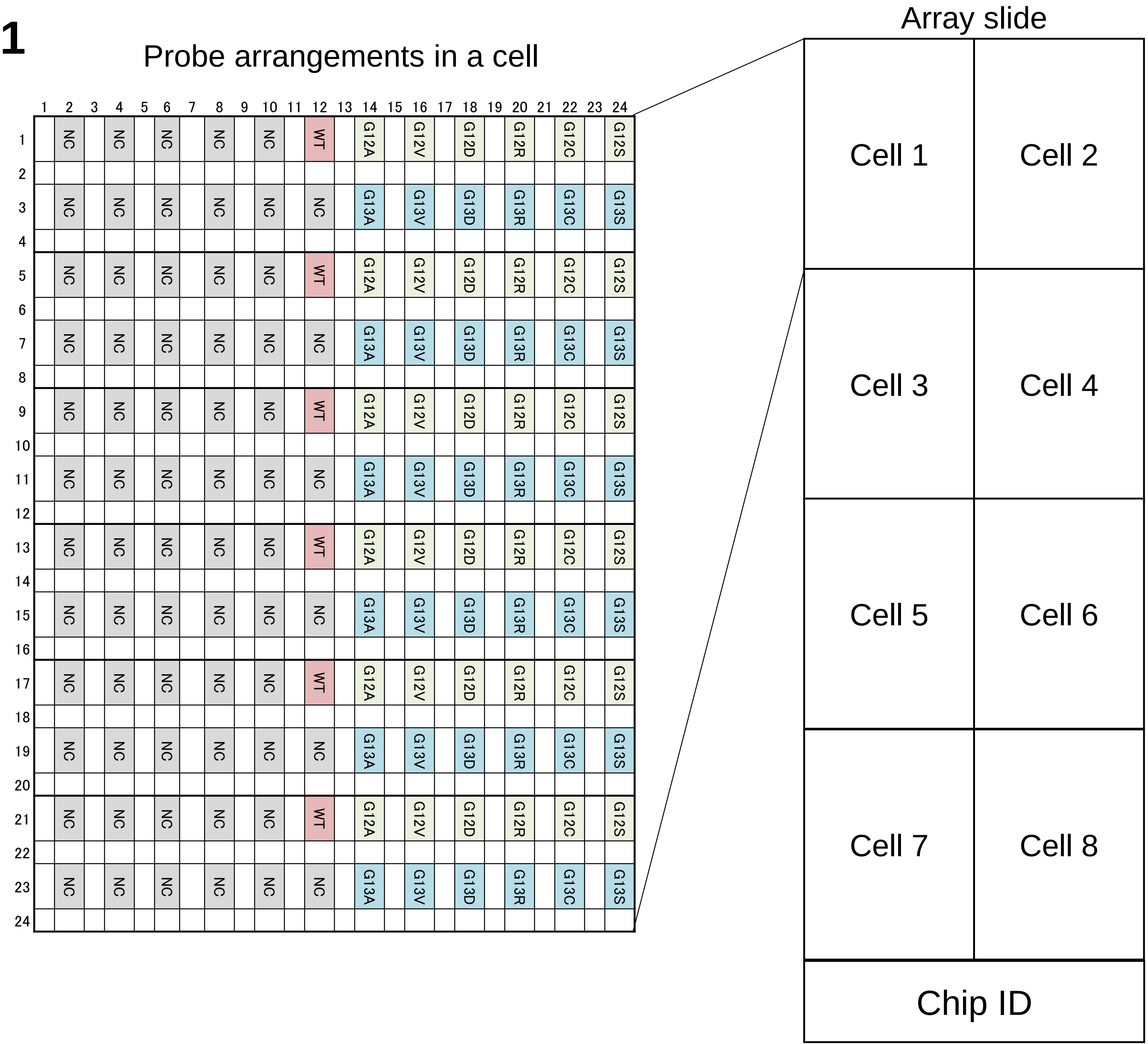

Probe arrangements in a cell
1 2 3 4 5 6 7 8 9 10 11 12 13 14 15 16 17 18 19 20 21 22 23 24
NC
WT
G12A
G12V
G12D
G12R
G12C
G12S
G13A
G13V
G13D
G13R
G13C
G13S
Array slide
Cell 1
Cell 2
Cell 3
Cell 4
Cell 5
Cell 6
Cell 7
Cell 8
Chip ID

Figure S2

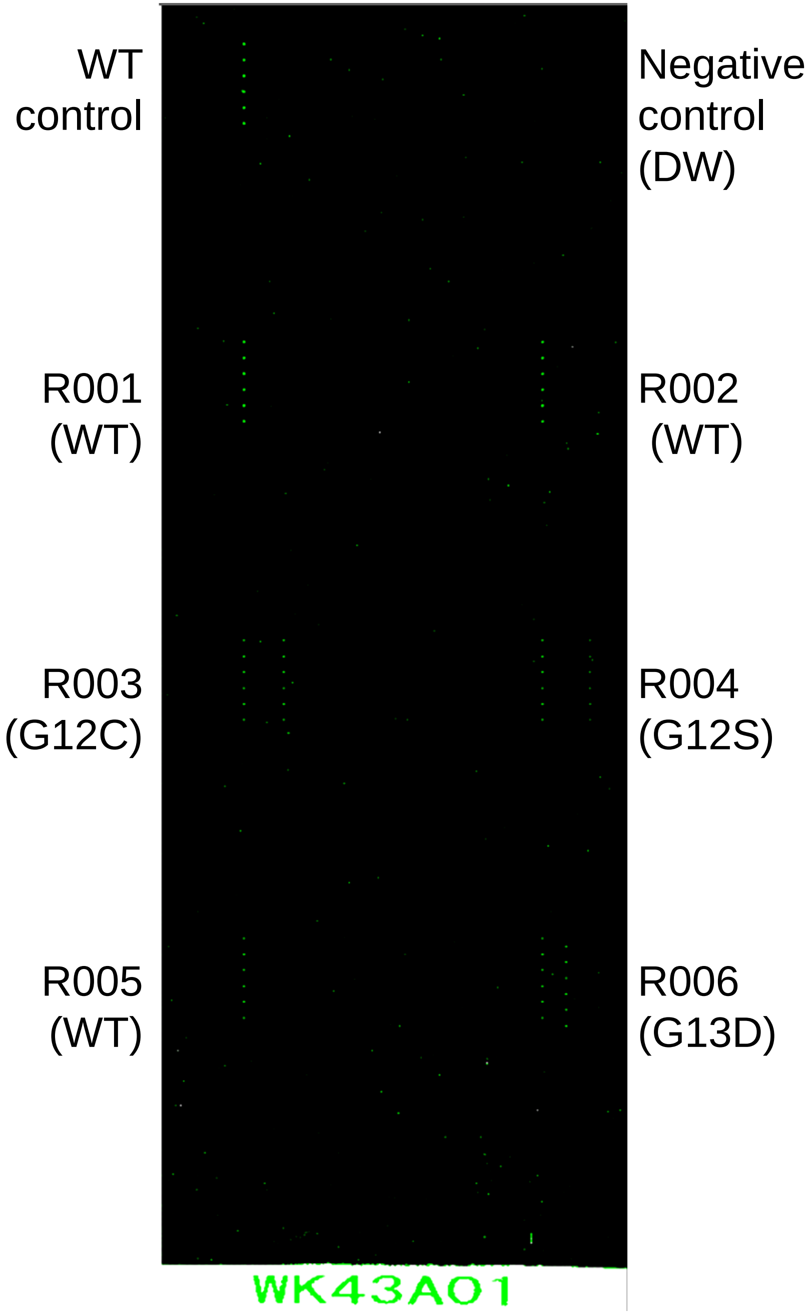

WT control
Negative control (DW)
R001 (WT)
R002 (WT)
R003 (G12C)
R004 (G12S)
R005 (WT)
R006 (G13D)
WK43A01
